# Molecular Cloning and Heterologous Expression of the Mitochondrial *ATP6* Gene from Kenaf (*Hibiscus cannabinus*) in Tobacco (*Nicotiana tabacum*)

**DOI:** 10.3390/genes16050479

**Published:** 2025-04-23

**Authors:** Bangbang Huang, Meiling Wei, Rongchang Wei, Wenhuan Hou, Xingfu Tang, Yanhong Zhao, Xiaofang Liao, Ruiyang Zhou

**Affiliations:** 1 College of Agriculture, Guangxi University, Nanning 530004, China; gxu14763770897@163.com; 2 Institute of Cash Crop, Guangxi Academy of Agricultural Sciences, Nanning 530007, Chinawenhuanhou@163.com (W.H.); xingfutang1018@163.com (X.T.); zhaoyanhong402@163.com (Y.Z.); 3 Soil and Fertilizer Work Station, Lingshan 535499, China; weimeiling1608@sina.com

**Keywords:** kenaf, cytoplasmic male sterility, *atp6*, construction of overexpression vectors, transformation

## Abstract

Background: The aim of this study was to develop a genetic transformation system to construct an overexpression vector for the mitochondrial gene *atp6* in tobacco, thereby providing a foundation to investigate the functional roles of mitochondrial genes in this species. Methods: A full-length coding sequence (CDS) of the *atp6* gene from a sterile line was cloned, along with the mitochondrial leader peptide sequence of *atp2-1* from tobacco, using cDNA from kenaf UG93A anthers as a template. An overexpression vector for plants was constructed by employing In-Fusion technology, and wild-type tobacco plants were transformed via *Agrobacterium*-mediated transformation. Transgenic tobacco plants were then subjected to resistance screening and PCR validation. Results: The overexpression vector PBI121-*atp2-1*-*atp6*-EGFP, which includeds the mitochondrial leader peptide sequence, was successfully constructed. PCR validation using two pairs of primers targeting different sites on the overexpression vector confirmed the stable expression of the target gene in six transgenic tobacco plants (H1, H3, H4, H5, H7, and H8) via both primer pairs. A phenotypic analysis and iodine–potassium iodide (I_2_-KI) staining of pollen grains from transgenic tobacco plants revealed the presence of shriveled and malformed pollen grains with reduced viability. These findings suggest that the *atp6A* gene, including the mitochondrial signal peptide, induces pollen abortion in tobacco. Conclusions: The genetic transformation system developed for the vector overexpressing the *atp6* mitochondrial gene from kenaf provides a valuable framework to investigate the molecular regulatory mechanisms underlying the role of the *atp6* gene in kenaf cytoplasmic male sterility (CMS).

## 1. Introduction

Kenaf (*Hibiscus cannabinus*) is an annual herbaceous fiber crop belonging to the Malvaceae family and *Hibiscus* genus. andIt is known for its drought tolerance, rapid growth, high biomass, and strong stress resistance [1]; it serves as a vital raw material for the hemp textile and paper-making industries because of its strong fibers, good moisture absorption, quick water dispersion, and resistance to corrosion and wear, and it has a broad range of applications [2,3]. Cytoplasmic male sterility (CMS) is an economical and effective pollination control system. The use of CMS hybrid seed production technology not only eliminates the cost of manual emasculation and reduces the environmental pollution caused by chemical-based male killing but also effectively increases the purity of large-scale seed production. This technology has been widely applied to crops such as rice, corn, kenaf, cotton, rapeseed, sorghum, and chili peppers [4,5]. Heterosis is the phenomenon in which heterozygotes are superior to both parents in one or more traits. The heterosis of kenaf is obvious [6]. The selection of CMS lines provides an important basis for the utilization of heterosis in kenaf. In 2002, Professor Zhou Ruiyang discovered a male sterile mutant in the wild-type UG93 progeny of kenaf whilste working in Hainan during the winter. Subsequent crosses with cultivated varieties as male parents revealed complete male sterility in certain F1 progeny, confirming that the mutant plant was a CMS type [7]. In 2003, the CMS line K03A, which is highly resistant to kenaf anthracnose, and its corresponding maintainer line K03B were successfully developed, achieving the trinity of kenaf CMS systems [8]. In 2008, the first hybrid kenaf variety, Hongyou No. 1, was bred with the CMS line, and the hybrid varieties obtained using the trinity method have become the primary varieties used in the Chinese hemp industry [9].

The *atp6* gene is a subunit gene of mitochondrial ATP synthase, which encodes part of the F0 component of the F0–F1 complex. Extensive research indicates that plant CMS is closely related to the mitochondrial gene *atp6*. Mutations in the *atp6* gene sequence lead to abnormalities in the synthesis of encoded amino acids and ultimately cause defects in ATP synthase function, leading to a lack of energy within cells, difficulty maintaining normal physiological functions, and male sterility in plants. Certain studies suggest that the interference of a single gene or multiple gene products in the function of mitochondrial F(0)F(1)-ATPase affects the energy-intensive process of pollen development [10]. The *atp6* gene plays a regulatory role in plant CMS;this was first discovered in corn. The cotranscription of the *rrn26* gene and part of the *atp6* gene in CMS-T results in the production of a toxic protein closely related to the mitochondrial inner membrane, leading to CMS in corn [11,12]. Similar findings have been reported for rapeseed, rice, and mustard. In the Pol-type CMS line of rapeseed, the cotranscription of *atp6/orf224* leads to CMS-Pol-type CMS [13,14]. In rice, the *orf79* gene in CMS-BT and the *orfH79* gene in CMS-HL are cotranscribed with the mitochondrial *atp6* gene, which encodes a toxic membrane protein similar to the N-terminus of COX1, also leading to CMS-HL and CMS-BT types of rice CMS [15]. Additionally, in mustard, the cotranscription product of *atp6/orf228* encodes a toxic protein, and the *atp6/orf263* gene encodes a membrane protein, leading to the CMS-Hau and CMS-Tour types of mustard CMS [16,17]. Research on the regulation of kenaf CMS by *atp6* has been conducted: Li Gang [18] used iTRAQ in 2009 to label mitochondrial proteins in the anthers of the kenaf maintainer line L23B and the CMS line L23A. They reported that the expression of *atp6* in the maintainer line was significantly higher than in the sterile line, suggesting that *atp6* plays a crucial role in kenaf anther development. In the homozygous sterile line P3A, the CDS of the *atp6* gene was observed to be 30 bp shorter than the maintainer line P3B. Based on the differences in the sequence characteristics of the CDS, existing CMS lines, maintainer lines, F1 and F2 generations, and 104 planting resources of kenaf were tested. The results confirmed that the 30 bp deletion in the sterile line was closely related to cytoplasmic male sterility in kenaf [19]. Liao [20] used Northern blot technology to analyze the transcripts of the *atp6* gene in the isonuclear homogametic UG93B and UG93A lines of kenaf, revealing that the sterile line had an additional 2.0 kb transcript compared with the maintainer line and that the expression of the *atp6* gene in the maintainer line was significantly higher than in the sterile line. *atp6* is clearly an important candidate factor regulating kenaf CMS.

The construction and transformation of *atp6* expression vectors are necessary for the study of gene function.Mitochondrial genes related to crop CMS must be fused with mitochondrial signal peptides to function [21,22]. Traditional expression vector construction methods mainly involve double enzyme digestion to link the target fragments, which results in many restrictions on the choice of enzyme cutting sites. First, a buffer suitable for both enzymes must be selected; otherwise, incomplete cutting of the vector may occur. The process is lengthier if distributed enzyme digestion is used. Second, the substrate DNA sequence must be analyzed for enzyme cutting sites before an expression vector is constructed via double enzyme digestion. If the substrate DNA sequence has unusable enzyme cutting sites or, conversely, if the substrate DNA sequence has all the enzyme cutting sites of the entire vector, then the double enzyme digestion method cannot be used to construct the expression vector [23]. Traditional double enzyme digestion methods have many limitations when used to construct more complex vectors, such as tissue-specific vectors, affecting the progression of the experiment. The construction of fusion expression vectors can efficiently express the target protein by fusing the target gene with other genes in the expression vector. This approach is conducive to the soluble expression of the target protein and facilitates the identification and tracking of the expression of the target protein. Our research group previously constructed an overexpression vector, pBI121-*atp6*-EGFP, which lacks a mitochondrial signal peptide and subsequently transformed both tobacco and *Arabidopsis thaliana* [20,24]. Prior studies have indicated that exogenous CMS genes in plants require guidance from signal peptides to effectively function within mitochondria [21,25]. In this study, we developed a novel overexpression vector, PBI121-atp2-1-*atp6*-EGFP—, which incorporateds a mitochondrial signal peptide. This vector was used to transform tobacco via the leaf disc methodto elucidate the regulatory mechanism of the *atp6* gene in plant fertility. In this study, kenaf UG93A anther cDNA was used as the template to clone the full-length CDS of the *atp6* gene via homologous cloning. The mitochondrial signal transit peptide *atp2-1* was subsequently recombined with the kenaf mitochondrial gene *atp6* using In-Fusion gene fusion technology to construct the PBI121-*atp2-1*-*atp6*-EGFP plant overexpression vector. *Agrobacterium*-mediated transformation was then used to transform wild-type tobacco, and transgene-positive plants were selected for resistance and verified using PCR of the target gene. These results are important for the establishment and functional study of the kenaf mitochondrial genetic transformation system.

## 2. Materials and Methods

### 2.1. Experimental Materials

In this study, we used the UG93A sterile line of kenaf as the starting material, which was grown at the experimental base of the College of Agriculture, Guangxi University. Anthers were harvested at the flowering stage, quickly frozen, and stored at −80 °C. The plant expression vector pBI121 and the wild-type tobacco K346 seeds were stored in the Plant Genetic Breeding Laboratory of the Comprehensive Building of the College of Agriculture, Guangxi University [24]. *Agrobacterium tumefaciens* EHA105 competent cells, *Escherichia coli* DH5α competent cells, high-fidelity enzymes, and reverse transcription kits were purchased from Beijing TransGen Biotech Co., Ltd. (Beijing, China). The restriction endonucleases KpnI and XbaI as well as M × Buffer, and T-vector ligation kits were obtained from Baori Doctor Biotechnology (Beijing) Co., Ltd. (Beijing, China). PCR product recovery kits, plasmid extraction kits, and standard DNA markers were acquired from Nanjing Vazyme Biotechnology Co., Ltd. (Nanjing, China). and Beijing Aidelai Biotechnology Co., Ltd. (Beijing, China). The primer synthesis was completed by Shenzhen Hua Da Gene Technology Co., Ltd. (Shenzhen, China) and bacterial liquid sequencing was performed by Shenggong Bioengineering (Shanghai) Co., Ltd. (Shanghai, China).

### 2.2. Experimental Methods

#### 2.2.1. Primer Synthesis

Primer sequences for the *atp6* gene, EGFP, and the upstream region of the pBI121 vector were designed using Premier 5.0 software. The details of the specific sequences are presented in Table 1.

#### 2.2.2. Construction and Transformation of the Multi-Fragment atp2-1-*atp6*-EGFP Fusion Gene Overexpression Vector

(1) Cloning of the Overexpression Target Fragment: Total RNA was extracted from kenaf UG93A anthers using a modified CTAB method and reverse transcribed into cDNA according to the instructions of the QuanShiJin reverse transcription kit. The cDNA served as the template to amplify the full-length coding sequence (CDS) of the *atp6* gene using the primer pair eMTS-*atp6* F/eMTS-*atp6* R. The mitochondrial transit peptide sequence *atp2-1* was amplified from tobacco K346 DNA preserved in our laboratory using the primer combination r*atp2-1*F/r*atp2-1*R [24].

(2) Fusion of Target Fragments and Amplification of the Fusion Fragment: Based on PCR for the fusion fragment, the molar ratio of the single fragments (*atp6* and *atp2-1*) was determined from their base pair counts. Under equimolar conditions, the total DNA amount required for the fusion PCR was set at 1000 ng, from which the DNA mass required for each single fragment was calculated (m*atp6* = 870 ng; m*atp2-1* = 130 ng). Using the formula V = m/c, the required amount of each gene fragment was determined based on the concentration of the recovered fragments. Fusion PCR was performed in a 50 μL reaction mixture as follows: 25 μL of 2 × Phanata Max Master Mix, 870 ng DNA*atp6*, 130 ng DNA*atp2-1*,and the addition of double-distilled water (ddH_2_O) to a 50 μL final volume. The PCR conditions to obtain the atp6-atp2-1 fusion PCR intermediate product were as follows: predenaturation at 95 °C for 5 min; 15 cycles of denaturation at 95 °C for 15 s, annealing at 60 °C for 20 s, and extension at 72 °C for 90 s; and a final extension at 72 °C for 5 min. The amplification of the fusion PCR intermediate product was performed in a reaction volume of 50 μL as follows: 25 μL of 2 × Phanata Max Master Mix, 2.5 μL fusion intermediate product DNA, 1.5 μL r*ATP2-1*-F, 1.5 μL rMTS-*atp6* R, and19.5 μL ddH_2_O. The PCR conditions were as follows: predenaturation at 95 °C for 5 min; 30 cycles of denaturation at 95 °C for 10 s, annealing at 60 °C (adjusted according to the primer annealing temperature) for 30 s, and extension at 72 °C for 90 s; and a final extension at 72 °C for 5 min. Finally, the fusion gene fragment PCR product was detected via electrophoresis on 1.0% agarose gel. The recombinant ligation product *atp6*-*atp2-1* was recovered using a DNA gel recovery kit, followed by ligation and transformation according to the QuanShiJin T-vector ligation kit protocol, and positive clones were sequenced.

(3) Target Vector Construction: The vector gene PBI121-FullHcpdil5-2a-EGFP preserved by our research group was modified. The plasmid was amplified and extracted for double digestion using the enzymes XbaI and KpnI. Double digestion was performed as follows: 2 μL of 10× buffer (M), 1 μLXbaI, 5 μLKpnI, 1 μg plasmid DNA, and the addition of ddH_2_O to a final reaction volume of 20 μL. The reaction was performed at 37 °C for 3 h. After the FullHcpdil5-2a gene was digested, the linear vector PBI121-EGFP was obtained, and the cut linear vector fragment was recovered using a DNA gel recovery kit. The double-digested target fragment *atp2-1*-*atp6* was ligated with the linear vector PBI121.The ligation reaction was performed as follows: 4 μL of the target fragment product, 5 μL Solution Ⅰ (BaoBio, Beijing, China), and 1 μL PBI121-EGFP vector. The reaction mixture was gently mixed the reaction was conducted at 16 °C for 7–8 h and then transformed into competent *E. coli* cells on ice. Finally, the successfully constructed expression vector was named PBI121-*atp2-1*-*atp6*-EGFP, abbreviated as the H overexpression vector gene.The overexpression vector was extracted and transformed into competent *Agrobacterium* EHA105 cells in preparation for the *Agrobacterium* infection of tobacco leaf discs.

(4) Detection and Sequencing of the Target Band: Eight white colonies were randomly picked and placed into 2 mL centrifuge tubes containing 1 mL of an LB liquid medium (with a final concentration of 0.1 mg/mL Amp). After incubation at 37 °C and 200 r/min for 12 h, the PCR detection of the bacterial mixture was performed according to the corresponding target gene amplification reaction program. PCR amplification products were detected via electrophoresis on 1.0% agarose gel, and positive bacterial bodies were identified by the presence of the target band. Each positive clone broth was divided into two portions on a superclean workbench, 150 µL of 60% glycerol was added to each portion and they were mixed well. Four positive clones were randomly selected, numbered, and sent to Shanghai Shenggong for sequencing. The remaining broth was stored at −80 °C for later use. The sequencing results were analyzed using DNAMAN and the NCBI database. The correct clones were selected for plasmid extraction and transformed into competent *Agrobacterium* EHA105 cells in preparation for the *Agrobacterium* infection of tobacco leaf discs.

(5) Transformation of Onion Epidermal Cells with the Overexpression Vector: The *Agrobacterium* strain PBI121-*atp2*-1-*atp6*-EGFP, which was successfully transformed with the target gene, was inoculated into 100 mL of an LB liquid medium supplemented with three antibiotics (50 mg/mL kanamycin, 100 mg/mL streptomycin, and 50 mg/mL rifampicin) and 100 mmol/L acetylsalicylic acid. Following a 48 h incubation at 28 °C under shaking conditions, the bacterial culture was centrifuged at 2800× *g* for 10 min to collect the cells. The cells were subsequently resuspended in an MS liquid medium containing 10 mmol/L MgCl_2_ and 100 mmol/L acetylsalicylic acid, and the OD600 of the bacterial suspension was adjusted to 0.6. The fresh middle layers of onion scales were immersed in 75% ethanol for 10 min. The samples were subsequently washed three times with sterile water, and the thick scales were excised using a sterile scalpel. A 1 cm^2^ square block was prepared on the inner epidermis of the concave surface of the onion. Forceps were used to carefully peel off the inner epidermal layer of the small square block, which was subsequently transferred into a bacterial suspension in an MS liquid medium and incubated for 20 min to allow infiltration. The bacterial suspension was filtered using filter paper, ensuring minimal retention of the liquid, and evenly spread onto Petri dishes containing a high-osmotic medium (MS + 30 g/L sucrose + 0.4 mol/L mannitol + 5.5 g/L agar; pH 5.8). The Petri dishes were sealed with parafilm and incubated in a growth chamber under a 16 h light/8 h dark cycle at 25 °C for 16 h of coculture. Following coculture, the onion epidermal pieces were retrieved and thoroughly rinsed with a sterile MS liquid medium to remove the adhered *Agrobacterium*. Microscope slides were prepared using the pressure plate method, and images were observed and captured using a fluorescence microscope.

(6) Transformation of Tobacco with the Overexpression Vector: The *Agrobacterium* transformation inoculum was transferred into 50 mL of a liquid medium (YEP + Kan (50 mg/L) + Rif (25 mg/L) + Str (25 mg/L)) and incubated at 28 °C with shaking at 220 rpm until the OD600 was approximately 0.6. The sample was centrifuged at 4000 rpm for 15 min, the supernatant was discarded, and the cells were resuspended in a sterile MS liquid medium to an OD600 of 0.3–0.5. Tobacco leaves were inoculated with the engineered *Agrobacterium* bacterial solution prepared at a concentration of 1.0. The precultured tobacco leaves were transferred to a 200 mL glass bottle and suspended in the *Agrobacterium*-enriched bacterial mixture for 10 min for infiltration. The tobacco leaves were transferred to filter paper to absorb the surface moisture of the leaves. Finally, the tobacco leaves were transferred to a coculture plate and cultivated in the dark at 25 °C for 3 days. The cocultured tobacco leaves were washed with sterile water and then washed with sterile water containing 500 mg/L cephalosporin. The tobacco leaves were dried on sterile blotting paper and then inoculated with a shoot induction medium. The tobacco leaves were then inoculated with a bud induction medium (MS + 6-BA [1 mL/L] + NAA [1 mL/L] + Kan + Cef + sucrose [30 g/L] + agar [6 g/L]; pH 5.8) and cultured at 26 °C for a photoperiod of 16 h light (2000 Lux)/8 h dark. Every 12 days, the tobacco leaf callus was subcultured once until adventitious buds grew. When the adventitious buds of the transgenic tobacco plants reached 2–3 cm, they were cut and transferred to a root induction medium to induce adventitious root formation. When the adventitious roots of the transgenic plantlets reached a length of 5–6 cm, they were transplanted for cultivation, and eventually, the transgenic tobacco was detected. The plantlets were grown in an artificial climate box under the following conditions in our laboratory: 26 ± 1 °C, 60% relative humidity (RH), and a long photoperiod of 16:8 (L/D) h in our laboratory. The culture soil used for transplanting was vermiculite/nutrient soil (2:1)and the pH of the nutrient soil was controlled at 5.5–6.5.

(7) Identification of Transgenic Plants: (i) The PCR detection of transgenic tobacco plants was performed when the transgenic tobacco tissue culture-generated plantlets had developed three leaves. The resistant transgenic tobacco tissue culture-generated plantlets were individually numbered to confirm whether they were positive. DNA was extracted from both wild-type tobacco and transgenic plants using the CTAB method. Specific primers for the *atp2-1*-*atp6* fusion fragment (r*atp2-1*F/eMTS-*atp6*R) and vector primers (PBI121-ZF/eMTS-*atp6*R; r*atp2-1*F/EGFP R) were used to prevent false positives. The corresponding DNA was amplified and detected using PCR, with wild-type tobacco serving as a negative control and the extracted PBI121-*atp2-1*-*atp6* overexpression vector gene plasmid serving as a positive control. The PCRs and programs were performed as previously described. (ii) The iodine–potassium iodide (I_2_-KI) staining method was used toidentify transgenic pollen grains. During the flowering stage, open anthers were collected, and several anthers were placed on a microscope slide using tweezers. A drop of a 1% I_2_-KI solution was added and the anthers were gently pressed with tweezers to release the pollen grains into the 1% I_2_-KI solution. After being stained for 5 min, a cover slip was applied, and the samples were observed and photographed using a microscope. The pollen grains were prepared for scanning electron microscopy (SEM) using the techniques of Liao et al. [20]. The acetolyzed pollen grains were suspended in 90% ethanol and then placed on a stub. The samples were sputter-coated with gold and then scanned and photographed using SEM (Tescan VEGA 3 LMU, Brno, Czech Republic).

## 3. Results

### 3.1. Cloning of the Target Genes

**Figure 1:**
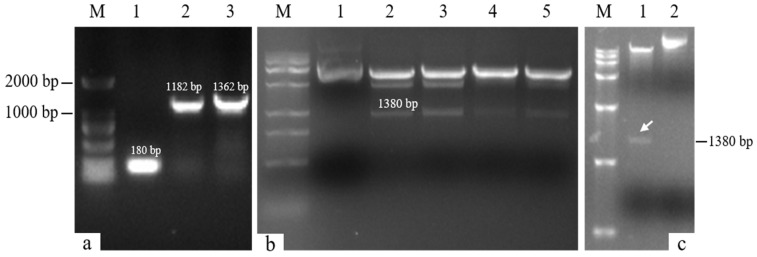
Fusion gene detection. (**a**) M: DL2000 DNA marker; Lane 1: atp2-1 gene; Lane 2: atp6 gene; Lane 3: atp2-1-atp6 fusion fragment. (**b**) M: DL15000 DNA marker; Lane 1: control PMD19-T-atp2-1-atp6 plasmid; Lanes 2~5: enzymatically digested fragment atp2-1-atp6 (1380 bp). (**c**) M: DL15000 DNA marker; Lane 1: double-digested fragment of the carrier gene plasmid PBI121-atp2-1-atp6-EGFP; Swimmer Lane 2: control plasmid PBI121-atp2-1-atp6-EGFP.

**Figure 2:**
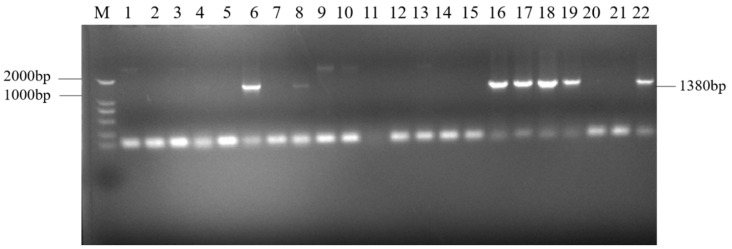
PCR products of the ligation of the fusion fragment to the PMD19-T- vector. Note: M: DNA marker (DL2000); Lanes 6, 8, 16–19, and 22: PMD19-T-atp2-1-atp6; Lanes 1–5, 7, 9–15, and 20–21: failure to connect.

The target single fragments *atp2-1* (180 bp) and *atp6* (1182 bp) were amplified using PCR. The target fragments were recovered and fused using a fusion PCR method to obtain the fused target fragment *atp2-1*-*atp6* (Figure 1a). The resulting fusion fragment was ligated with the PMD19-T vector and transformed. The bacterial liquid PCR results indicated that a total of seven positive clones containing the fusion target fragment were amplified (Figure 2). Three positive clones were randomly selected for bacterial liquid sequencing, and all yielded the correct target sequence. The PMD19-T-*atp2-1*-*atp6* fusion fragment was amplified, and the plasmid DNA was extracted and then double-digested using XbaI and KpnI. Gel electrophoresis confirmed the presence of the correctly fused *atp2-1*-*atp6* gene fragment (Figure 1b).

### 3.2. Results of the Overexpression Vector Construction

**Figure 3:**
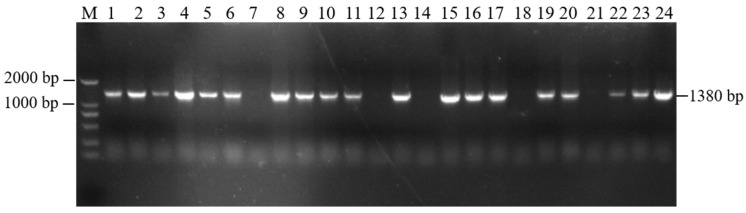
PCR detection of the target fragment bacterial mixture. M—DNA molecular quality standard (DL2000).

The recovered overexpression fusion gene fragment *atp2-1*-*atp6* was ligated with the linear PBI121-EGFP vector fragment to obtain the recombinant vector PBI121-*atp2-1*-*atp6*-EGFP. The PCR revealed a total of 19 positive clones whose band sizes were consistent with the corresponding target fragments (Figure 3). A random sequencing comparison was performed on the aforementioned overexpression vector gene bacterial mixtures (PBI121-*atp2-1*-*atp6*-EGFP), followed by plasmid extraction and double-enzyme digestion identification to avoid false positives from single colonies carrying this overexpression vector during the construction and transformation process. As shown in Figure 1c, when the overexpression vector PBI121-*atp2-1*-*atp6*-EGFP was used as a positive control, the results revealed that the target fragment obtained from double enzyme digestion was approximately the same size as the ligated target fragment (*atp2-1*-*atp6*); this further confirmed that the overexpression vector was correctly constructed and could be used for the subsequent *Agrobacterium* transformation.

### 3.3. Analysis of Subcellular Localization in Onion Epidermal Cells

**Figure 4:**
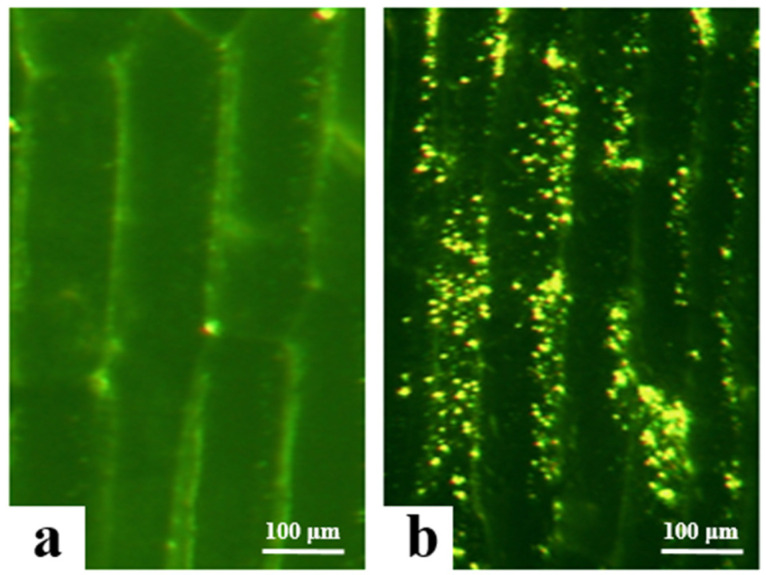
Results of the transformation of onion epidermal cells with the EGFP transient overexpression vector. (**a**) transgene with PBI121-EGFP; (**b**) transgene with PBI121-*atp2-1-atp6*-EGFP.

In this study, the successfully transformed *Agrobacterium* carrying the overexpression vectors (PBI121-EGFP and PBI121-*atp2-1*-*atp6*-EGFP) was used to infect and transform onion epidermal cells to determine the initial subcellular localization of the *atp6* protein. Under a fluorescence electron microscope, the empty vector PBI121-EGFP produced green fluorescence signals only on the cell membrane, whereas the target fragment vector PBI121-*atp2-1*-*atp6*-EGFP exhibited substantial expression within the cytoplasm of onion epidermal cells, with intense and numerous green fluorescence signals (Figure 4). Owing to the presence of the tobacco mitochondrial signal peptide sequence *atp2-1* in the PBI121-*atp2-1*-*atp6*-EGFP fusion vector, this sequence could transport *atp6* to the mitochondria. Concurrently, the green fluorescent protein EGFP exhibited spontaneous fluorescence, which enabled the determination of the location at which the protein encoded by the gene in this vector was expressed within the cell. These results indicate that the *atp6* protein from the CMS line of kenaf was stably expressed in the mitochondria.

### 3.4. Agrobacterium-Mediated Transformation of Tobacco Leaf Discs

**Figure 5:**
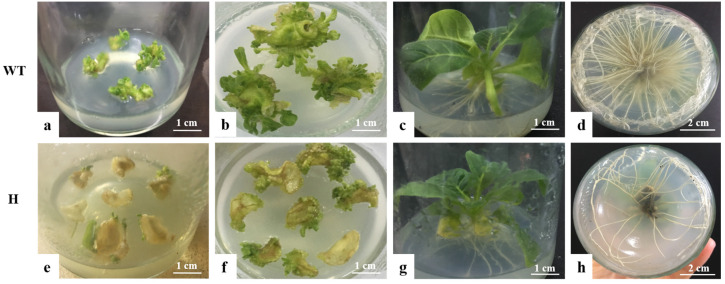
Bud induction culture of tobacco leaf discs. (**a**–**d**) wild-type tobacco (CK); (**e**–**h**) transgenic tobacco (H). (**a**) Differentiation of calli in wild-type tobacco. (**b**) Adventitious buds of wild-type tobacco. (**c**) Root induction growth of wild-type tobacco. (**d**) Root growth of wild-type tobacco. (**e**) Differentiation of calli in transgenic plants (H). (**f**) Adventitious buds of transgenic plants (H). (**g**) Root induction growth of transgenic plants (H). (**h**) Root growth of transgenic plants (H).

The overexpression vector PBI121-*atp2-1*-*atp6*-EGFP was subsequently transformed into *Agrobacterium*, and laboratory-preserved tobacco K346 was used as the transgenic material. The *Agrobacterium* infection of leaf discs was employed to transform the tobacco, with wild-type tobacco leaf discs not infected with *Agrobacterium* serving as the negative control (CK). The overexpression vector containing the mitochondrial signal transit peptide *atp6*, PBI121-*atp2-1*-*atp6*-EGFP, was abbreviated as H. The bud induction and root induction stages of tobacco leaf discs are shown in Figure 5. During the bud induction period, the wild-type tobacco leaf discs (negative control) exhibited faster sprouting with numerous bud points (Figure 5b). After the tobacco leaf discs were treated with PBI121-*atp2*-1-*atp6*-EGFP(H)-expressing *Agrobacterium*, they were placed in a medium containing the antibiotics kanamycin and rifampicin for screening as well as the cultivation of resistant buds. Compared with the control plants, the resistant transgenic H plants sprouted more slowly and presented fewer bud points (Figure 5f). These findings indicated that the acquisition rate of resistant transgenic tobacco buds was lower during the bud induction stage. The growth status of wild-type tobacco plants without antibiotics added to the root culture medium was used as the control during the rooting induction stage of tobacco cultivation (Figure 5c). The roots grew faster in the control group, and more, denser roots were observed (Figure 5d). Concurrently, the transgenic tobacco H plants in the root culture media supplemented with kanamycin and rifampicin (Figure 5g) grew more slowly overall than the control plants. Compared with the control plants, the root growth speed of the transgenic H plants (Figure 5h) was relatively slower, and the number of roots was smaller.

### 3.5. Identification of Positive Transgenic Tobacco Plants

**Figure 6:**
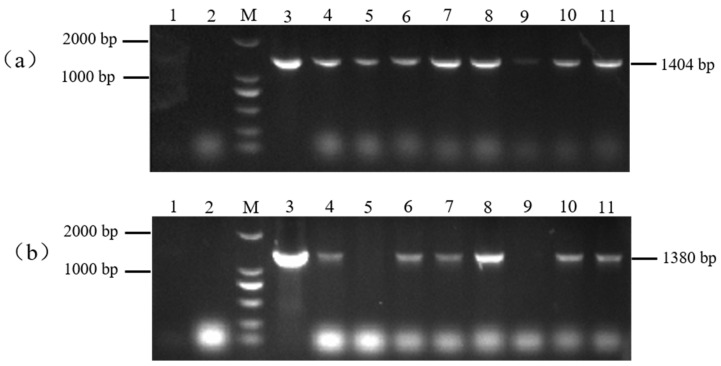
Identification of transgenic (PBI121-atp2-1-atp6-EGFP) tobacco materials. (**a**) M: DNA marker (DL2000); Lane 1: H_2_O; Lane 2: WT; Lane 3: PBI121-atp2-1-atp6-EGFP (1404 bp); Lanes 4–11: identification of transgenic plants (PBI121-atp2-1-atp6-EGFP (1404 bp)). (**b**) M: DNA marker (DL2000); Lane 1: H_2_O; Lane 2: WT; Lane 3: PBI121-atp2-1-atp6-EGFP (1380 bp); Lanes 4–11: identification of transgenic plants (Lanes 4–8 and 10–11: transgenic plants of PBI121-atp2-1-atp6-EGFP (1380 bp)).

The resistant plants (H: PBI121-*atp2-1*-*atp6*-EGFP) obtained from the selection were subjected to PCR detection with the primer combinations PBI121 F/*atp6* R and r*atp2-1*F/eMTS-*atp6* R to screen for the plants. As shown in Figure 6, with the DNA from wild-type tobacco (WT) plants and H_2_O as negative controls and the overexpression vector plasmid DNA as a positive control, a total of six T0 generation single positive PBI121-*atp2-1*-*atp6*-EGFP transgenic tobacco plantlets were obtained through kanamycin resistance selection and PCR amplification with two specific primer pairs (Lanes 4, 6, 7, 8, 10, and 11, with the corresponding plant abbreviations H4, H6, H7, H8, H10, and H11).

### 3.6. Observations of the Phenotypic Traits and Fertility of Transgenic Tobacco

**Figure 7:**
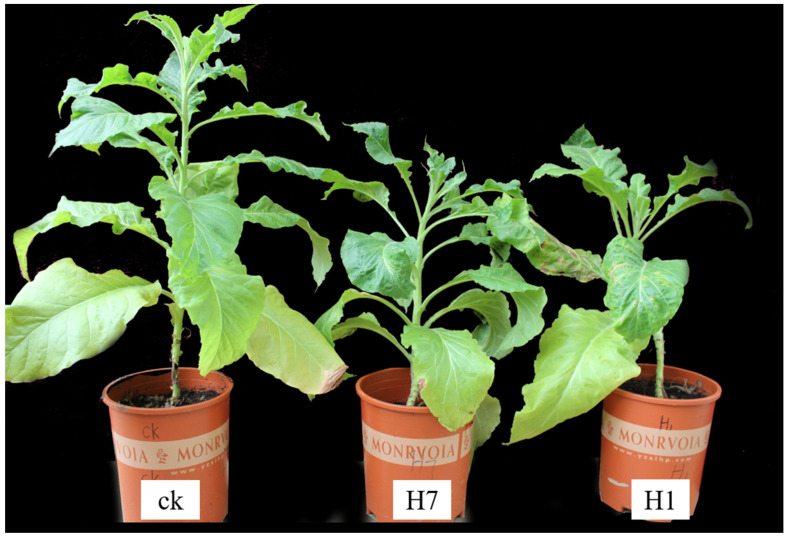
Comparison of the growth of wild-type and transgenic tobacco (H: PBI121-*atp2-1-atp6*-EGFP). The first plant (from left to right) is the wild-type plant plants H7 and H1 are transgenic tobacco plants.

**Figure 8:**
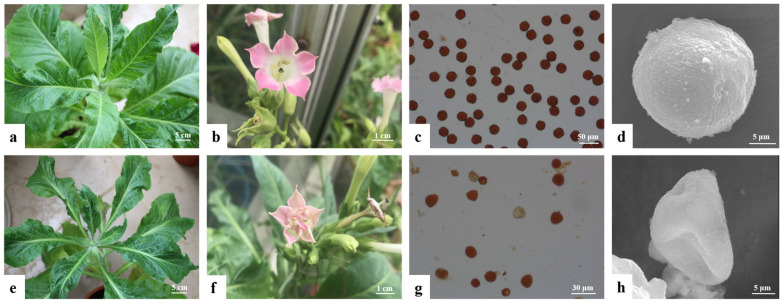
Comparison of the leaves and fertility of wild-type and transgenic tobacco plants. (**a**–**d**): wild-type tobacco; (**e**–**h**): transgenic H (PBI121-atp2-1-atp6-EGFP) tobacco; (**a**): wild-type tobacco plant; (**b**): wild-type tobacco flower; (**c**): I_2_-KI staining of wild-type tobacco pollen grains; (**d**): pollen grain of the wild-type plant; (**e**): transgenic H plant; (**f**): transgenic H tobacco flower; (**g**): I_2_-KI staining of transgenic tobacco (H) pollen grains; (**h**): pollen grains of transgenic H plants.

During the observations and analysis of the obtained positive transgenic tobacco plants, the overall growth conditions of the H1 and H7 transgenic tobacco plants (PBI121-*atp2-1*-*atp6*-EGFP) were significantly weaker and shorter within the same growth period than the wild-type tobacco and the control group, with more pronounced phenotypic differences (Figure 7). Positive transgenic tobacco plants also presented substantial differences in their malformed leaves compared with wild-type tobacco plants. The wild-type tobacco leaves (Figure 8a) and the transgenic tobacco H leaves (Figure 8e) were significantly different; the wild-type leaves were larger overall with normal and clear venation, whereas the transgenic tobacco plant H leaves were markedly different; They were thicker, smaller, and malformed with twisted venation. The leaf color of the transgenic plants was darker than the wild-type plants, and the leaf shape resembled a wedge shape. Based on a comparison between the wild-type and transgenic tobacco H, it could be concluded that the transgenic H line (PBI121-*atp2-1*-*atp6*-EGFP) showed very distinct phenotypic differences.

Comparative observation of the floral organs of the wild-type and transgenic tobacco revealed that the wild-type tobacco had a normal petal shape, pink color, and complete stamen development, with anthers capable of normal dehiscence and pollen dispersal (Figure 8b). In contrast, the transgenic tobacco plants (H) presented malformed flower growth andthe flowers were smaller than those of the control, with malformed and incomplete petals and a lighter coloration. The stamens adhered to the incomplete petals, and the anthers produced very little pollen upon dehiscence (Figure 8f). A further fertility assessment of the transgenic tobacco pollen grains using I_2_-KI staining revealed that the wild-type tobacco pollen grains stained normally (Figure 8c), whereas the transgenic tobacco (H) pollen grains did not darken because of iodine–potassium iodide staining, indicating a lack of staining (Figure 8g). These findings suggest that the pollen grains of the H transgenic tobacco had weak or no activity. Anoticeable difference in the size and quantity of pollen grains was observed between the wild-type and transgenic tobacco. The wild-type tobacco pollen grains were rounder, larger, and greater in quantity, whereas the transgenic tobacco pollen grains presented a few deformities, were smaller, and were fewer in number. An examination of the iodine–potassium iodide solution using a microscope and electron microscopy revealed that the pollen grains of the transgenic tobacco (H) had an abnormal morphology and poor development. These results indicate that the kenaf *atp6* sterility gene had a significant regulatory effect on the fertility of the tobacco.

## 4. Discussion

In this study, we cloned the *atp6* gene sequence using cDNA from the UG93A male-sterile material of kenaf as a template and constructed a plant expression vector containing a mitochondrial transit peptide, P35S::MTS-EGFP-*atp6* (PBI121-*atp2-1*-*atp6*-EGFP), using In-Fusion gene fusion technology [22]. Compared with traditional multifragment ligation methods, this technology is characterized by its speed, efficiency, and simplicity. This approach not only saves time and cost in experimental research when constructing vector genes but also avoids the use of various enzymes in the process It also overcomes the inherent limitations of cloning genes [23,26].

The construction of the plant expression vector P35S::MTS-EGFP-*atp6* (PBI121-*atp2-1*-*atp6*-EGFP) involved the fusion of two DNA fragments from different sources, which presented challenges because of the significant difference in the base pair sequence lengths of the genes (*atp2-1* and *atp6*). The *atp6* sequence, which is 1200 bp long, is a relatively large fragment, whereas the *atp2-1* sequence, which is 180 bp long, is considered to be too short. Using conventional enzymatic digestion and ligation methods to locate suitable restriction sites for longer fragments is difficult; the use of short *atp2-1* fragments also increases identification complexity [27]. The ligation process can also introduce unrelated and unnecessary restriction site base sequences. To address these issues, we employed fusion PCR technology in this study [28] to amplify gene fragments from different sources using primers with complementary ends, allowing the fusion and connection of the two fragments. Specifically, complementary primer sequences were designed at the 3′ end of the *atp2-1* transit peptide sequence and the 5′ end of the *atp6* gene, successfully fusing these two gene fragments. This approach also overcame the limitations of traditional PCR techniques [29]. After transformation into a T-vector for sequencing, the target fragment was excised using double enzymatic digestion and then recombined with the linear plasmid PBI121-EGFP to construct the recombinant vector. The successful construction of the overexpression vectors was confirmed using sequencing and verification via double enzymatic digestion. This method is a powerful, innovative tool for the construction of overexpression vectors related to kenaf CMS genes.

In this study, we constructed the P35S::MTS-*atp6*-EGFP overexpression vector by fusing the mitochondrial leader peptide with the target gene This approachdifferedfrom that of Wei Meiling [24], who directly constructed the *atp6* overexpression vector without fusing the mitochondrial leader peptide to study its function. In this study, initial subcellular localization in onion epidermal cells revealed that the *atp6* protein, under the guidance of the transport signal peptide *ATP2-1*, was concentrated in the mitochondria of the onion. The further transformation of the overexpression vector in tobacco and the successful acquisition of positive transgenic plants could provide a theoretical foundation to understand the molecular mechanism by which *atp6* regulates kenaf CMS. This finding could also haves significant implications for innovation in kenaf germplasm research and the utilization of hybrid vigor.

## 5. Conclusions

In this study we developed an *atp6*A overexpression vector incorporating a mitochondrial presequence via gene fusion and successfully transformed tobacco, resulting in the generation of two positive transgenic tobacco lines. Subcellular localization studies in onion epidermal cells confirmed the mitochondrial targeting of *ATP6*A. The phenotypic analysis and I_2_-KI staining of the transgenic tobacco pollen grains revealed a shrunken and deformed morphology with reduced viability, suggesting that *atp6*A with a mitochondrial presequence could induces pollen abortion in tobacco. These findings lay a theoretical foundation to understand the molecular mechanisms underlying *atp6*-regulated cytoplasmic male sterility (CMS) in kenaf and reveal significant implications for kenaf germplasm innovation and the exploitation of heterosis.

## Figures and Tables

**Table 1:** Primers used in this study.

Primer Names	Primer Sequences (5′-3′)
*ratp2-1* F:	tctagaATGGCTTCTCGGAGGCTTCTCGC
*ratp2-1* R:	AGTCCTTTTCATTGCCGCTGCGGAGGTAGCGTA
eMTS-*atp6* F:	CAGCGGCAATGAAAAGGACTATGTTTGTAAATATCATG
eMTS-*atp6* R:	ggtaccATGAAGATTTATAGCATCATTTAAGTAAATACAG
PBI121 F:	GGAGAGAACACGGGGGACTCTAGA

The sequences of the Xba I and Kpn I restriction enzymes are denoted using lowercase letters, whereas the 5′ homologous sequence of *atp6* and the 3′ homologous sequence of *ATP2-1* are indicated by underlined letters.

## Data Availability

The original contributions presented in this study are included in the article. Further inquiries can be directed to the corresponding authors.
